# MicroRNA-383-5p inhibits the progression of gastric carcinoma via targeting HDAC9 expression

**DOI:** 10.1590/1414-431X20198341

**Published:** 2019-07-29

**Authors:** Gang Xu, Na Li, Yan Zhang, Jinbiao Zhang, Rui Xu, Yanling Wu

**Affiliations:** Department of Oncology, Chinese PLA No.148 Hospital, Zibo, Shandong, China

**Keywords:** MicroRNA-383-5p, Gastric cancer, HDAC9, Post-transcriptional regulation

## Abstract

MicroRNAs (miRNAs), as post-transcriptional regulators, have been reported to be involved in the initiation and progression of various types of cancer, including gastric cancer (GC). The present study aimed to investigate the role of miR-383-5p in gastric carcinogenesis. Cell viability was analyzed using CCK-8 kit. Annexin V-fluorescein isothiocyanate/propidium iodide double staining was used to evaluate cell apoptosis. The expression levels of miR-383-5p and histone deacetylase 9 (HDAC9) mRNA in GC tissues and cell lines were analyzed using RT-qPCR. The protein expression of HDAC9 was detected by western blotting. We found that HDAC9 was up-regulated and miR-383-5p was down-regulated in GC tissues and cell lines. High HDAC9 expression or low miR-383-5p expression was closely related to poor prognosis and metastasis in GC patients. HDAC9 knockout or miR-383-5p mimics led to growth inhibition and increased apoptosis in AGS and SGC-7901 cells. More importantly, we validated that miR-383-5p as a post-transcriptional regulator inhibited HDAC9 expression and was inversely correlated with HDAC9 expression in GC tissues. miR-383-5p had the opposite effects to HDAC9 in gastric carcinogenesis. miR-383-5p played an important role in gastric carcinogenesis, and it is one of the important mechanisms to regulate oncogenic HDAC9 in GC, which might be helpful in the development of novel therapeutic strategies for the treatment of GC.

## Introduction

The incidence and mortality of gastric cancer (GC) have been increasing dramatically and rank second behind lung cancer in China (1). It is estimated that there will be about 679,100 new diagnoses and approximately 498,000 deaths from GC in 2015, and the incidence and mortality of GC are expected to account for 15.8 and 17.7%, respectively, of all cancer cases (1). Global cancer statistics reveal that incidence and mortality rank fourth and third, respectively, worldwide (2). Environmental factors, such as smoking and *Helicobacter pylori* infection, and genetic alterations are considered the major risk factors for the carcinogenesis of GC (3–5). Numerous studies reveal that multiple signaling pathways, including nuclear factor kappa B, phosphatidylinositol 3-kinase/protein kinase B, mitogen-activated protein kinase, methylation, and histone acetylation, contribute to the progression of GC (6–10). However, the underlying molecular mechanisms have not been completely clarified in this process.

Histone deacetylase 9 (HDAC9) belongs to HDAC family and catalyzes deacetylation of acetylated lysine residues on histone proteins (11). In the pathological process of cancer, HDAC9, as an oncogene, confers migratory, invasive, and angiogenic potential in various malignancies, including breast cancer, oral squamous cell carcinoma (OSCC), lymphoma, and medulloblastoma (12–15). Moreover, over-expression of HDAC9 is frequently associated with tumor progression and poor prognosis (16). In human GC, HDAC9 mutation is potentially involved with GC peritoneal carcinomatosis (17). However, the functions of HDAC9 during gastric tumorigenesis are still unclear.

MicroRNAs (miRNAs) represent a class of non-coding RNAs and are characterized by small, non-coding, and single-stranded RNAs (18–25 nucleotides), which regulate gene expression through sequence-specific interaction with its 3′-untranslated regions (3′-UTRs) (18). miRNAs have recently emerged as a novel class of post-transcriptional regulators in a variety of biological processes, including tumorigenesis (18). In the context of GC, characteristic miRNA signatures are closely associated with cell growth, migration, invasion, epithelial-mesenchymal transition, chemotherapy-resistance, and recurrence prediction (19–21). Recent studies have uncovered that miR-383 serves as a tumor suppressor in various cancers, including GC, by modulating multiple target genes (22,23). However, the role of miR-383-5p-regulated HDAC9 in GC has not been reported. Therefore, the present study explored whether miR-383-5p as a post-transcriptional regulator was involved in the progression of GC via modulation of HDAC9.

Here, we first detected the expression levels of miR-383-5p and HDAC9 in sixty-three pairs of GC and adjacent non-tumorous tissues. Subsequently, we investigated whether miR-383-5p or HDAC9 could serve as an independent factor for predicting survival prognosis of patients with GC. Bioinformatics algorithms and luciferase reporter analysis were performed to evaluate whether HDAC9 was a direct target of miR-383-5p. *In vitro* experiments of HDAC9 loss-of-function or miR-383-5p gain-of-function were used to explore the roles of HDAC9 and miR-383-5p, respectively, on GC cell proliferation and apoptosis. Furthermore, we found that miR-383-5p might serve as a tumor suppressor regulator of gastric tumorigenesis via post-transcriptional repression of HDAC9.

## Material and Methods

### Sample collection

Sixty-three pairs of GC tissues and corresponding non-tumorous tissues were collected from patients in the Department of Oncology of Chinese PLA No.148 Hospital (China) between Jan 2010 and Jan 2013. Both GC and non-tumorous tissue were histologically confirmed, and all samples were stored in an ultra-low temperature refrigerator (–80°C; Thermo Fisher Scientific, Inc., USA) for further experiments. Patients did not receive chemotherapy or radiotherapy prior to surgery. This study was approved by the Ethics Committee of the Chinese PLA No.148 Hospital (China; Approval No. C2010A0125).

### Cell culture

Human normal gastric epithelial cell line GES-1 and five GC cell lines (AGS, SGC-7901, MGC-803, HGC-27, and BGC-823) were obtained from the American Type Culture Collection (ATCC; USA). Cells were cultured in Dulbecco's modified Eagle's medium (DMEM; Invitrogen, USA) with 5% fetal bovine serum (Thermo Scientific HyClone, China), 5% CO_2_, 95% air in a humidified incubator (Thermo, USA).

### Plasmid constructs and cell transfection

Short hairpin RNA (shRNA) was designed to specifically target HDAC9 using shRNA design tools (http://rnaidesigner.thermofisher.com/rnaiexpress/). We verified if the designed shRNA targeted only the HDAC9 using BLAST (http://blast.ncbi.nlm.nih.gov/Blast.cgi). ShRNA-NC and sh-HDAC9 were synthesized by RiboBio (China).

The miR-383-5p mimic was synthesized by Ambion (Invitrogen) and transfected into AGS and SGC-7901 cells to a final concentration of 100 nM using Lipofectamine 2000 (Invitrogen; Thermo Fisher Scientific, Inc.) for 48 h at 37°C according to the manufacturer's protocol. The pre-miR-Con control sequences were purchased from Ambion (Invitrogen) and used as a negative control.

### CCK-8 assay

After transfection with shRNA-NC or sh-HDAC9, pre-miR-Con or miR-383-5p mimics, AGS and SGC-7901 cells (1×10^4^) were seeded in the 96-well plate for 24, 48, and 72 h, and cell viability was measured using CCK-8 Cell Proliferation/Viability Assay Kit (Japan). Absorbance was recorded at 450 nm using Elx800 Reader (Bio-Tek Instruments Inc., USA).

### Apoptosis assay

After transfection with shRNA-NC or sh-HDAC9, pre-miR-Con or miR-383-5p mimics, cell apoptosis assay was performed by flow cytometry analysis. Annexin V-FITC/PI apoptosis detection kit was purchased from Invitrogen. The samples were analyzed using a flow cytometer (FACScan, BD Biosciences, USA) and analyzed by CELL Quest 3.0 software (BD Biosciences).

### Luciferase reporter gene assay

The wild-type (WT) or mutant-type (MT) 3'-UTR of HDAC9 were synthesized by RiboBio (China) and inserted into the multiple cloning sites of the luciferase-expressing pMIR-REPORT vectors (Ambion; Thermo Fisher Scientific, Inc.), which were transfected into AGS and SGC-7901 cells with Lipofectamine 2000 (Invitrogen; Thermo Fisher Scientific, Inc.) for 48 h at 37°C according to the manufacturer's protocol. The luciferase activity was measured using the Dual Luciferase Reporter^®^ Assay System (Promega, USA) on a Luminoskan^TM^ Ascent Microplate Luminometer (Thermo Fisher Scientific).

### Reverse transcription-quantitative polymerase chain reaction (RT-qPCR)

Total RNA was extracted using TRIzol^®^ (Invitrogen; Thermo Fisher Scientific, Inc.), according to the manufacturer's protocol. RT was performed using TaqMan^®^ reverse transcription kit (Applied Biosystems; Thermo Fisher Scientific, Inc.), according to the manufacturer's protocol. miR-383-5p was detected using TaqMan^®^ MicroRNA assay (Applied Biosystems; Thermo Fisher Scientific, Inc.), according to the manufacturer's protocol. U6 small nuclear RNA was used as an endogenous control.

In addition, 2 μg total RNA was used to synthesize cDNA with moloney murine leukemia virus reverse transcriptase (Invitrogen; Thermo Fisher Scientific, Inc.), according to the manufacturer's protocol. RT-qPCR was performed using the Applied Biosystems 7300 Real-Time PCR system (Applied Biosystems; Thermo Fisher Scientific, Inc.) with the TaqMan^®^ Universal PCR Master Mix (Thermo Fisher Scientific, Inc.). The relative mRNA expression levels were calculated using the 2^-ΔΔCq^ method (24) and normalized to glyceraldehyde 3-phosphate dehydrogenase (GAPDH). The primers were synthesized by Invitrogen (Thermo Fisher Scientific, Inc.), and sequences were as follows: HDAC9 forward 5′-AGCCCATCTCACCTTTAGACC-3′ and reverse 5′-ATTGCTTCTCACGGACAACAG-3′; GAPDH forward 5′-ACAGGGGAGGTGATAGCATT-3′ and reverse 5′-GACCAAAAGCCTTCATACATCTC-3′.

### Western blotting

Proteins were extracted using radioimmunoprecipitation assay buffer (Beyotime Institute of Biotechnology, China). Protein concentration was measured using the Bicinchoninic Acid kit for protein determination (cat. No. BCA1-1KT; Sigma-Aldrich; Merck KGaA, Germany). Western blotting was conducted as previously described (13). The primary antibody of HDAC9 was purchased from Abcam (dilution: 1:1000; UK). Subsequently, the membranes were incubated with the appropriate horseradish peroxidase-conjugated secondary antibody (dilution: 1:10000; Santa Cruz Biotechnology, USA) at room temperature for 2 h and visualized by chemiluminescence (Thermo Fisher Scientific, Inc.). Signals were analyzed with Quantity One^®^ software version 4.5 (Bio Rad Laboratories, Inc., USA). β-actin (cat. No. sc-130065; dilution: 1:2000; Santa Cruz Biotechnology) was used as the control antibody.

### Establishment of tumor xenografts in mice

After transfecting pre-miR-Con or miR-383-5p mimics into AGS cells (1×10^7^ cells per 0.1 mL), cells were implanted subcutaneously into 4-week-old castrated male nude mice (n=12, Beijing HFK Bio-Technology. Co., LTD., China). Tumor volume and weight were measured when mice were sacrificed at week 6 after cell implantation. All the experimental protocols were approved by the Animal Care and Use Committee of Chinese PLA No.148 Hospital (China). The animal experiment was approved by the Ethics Committee of the Chinese PLA No.148 Hospital (China; Approval No. C2010A0125).

### Statistical analysis

Data are reported as means±SE. Statistical analysis was performed using IBM SPSS Statistics Version 19.0 (SPSS Inc., USA) and GraphPad Prism Version 7.0 (GraphPad Software, Inc., USA). Student's *t*-test was used to analyze two-group differences. Inter-group differences were analyzed by one-way analysis of variance, followed by a *post hoc* Tukey test for multiple comparisons. Pearson χ^2^ tests were used to evaluate differences between the clinical characteristics and HDAC9 or miR-383-5p expression levels in GC patients. Univariate and multivariate Cox proportional hazards regression analyses were used to test for independent prognostic factors. Spearman's rank analysis was used to identify the correlation between the expression levels of miR-383-5p and HDAC9. Survival rates were calculated using the Kaplan-Meier method with the log-rank test applied for comparison. P values less than 0.05 were considered to indicate a statistically significant difference.

## Results

### HDAC9 was up-regulated in GC tissues and cell lines, and was associated with poor prognosis

**Figure 1.**
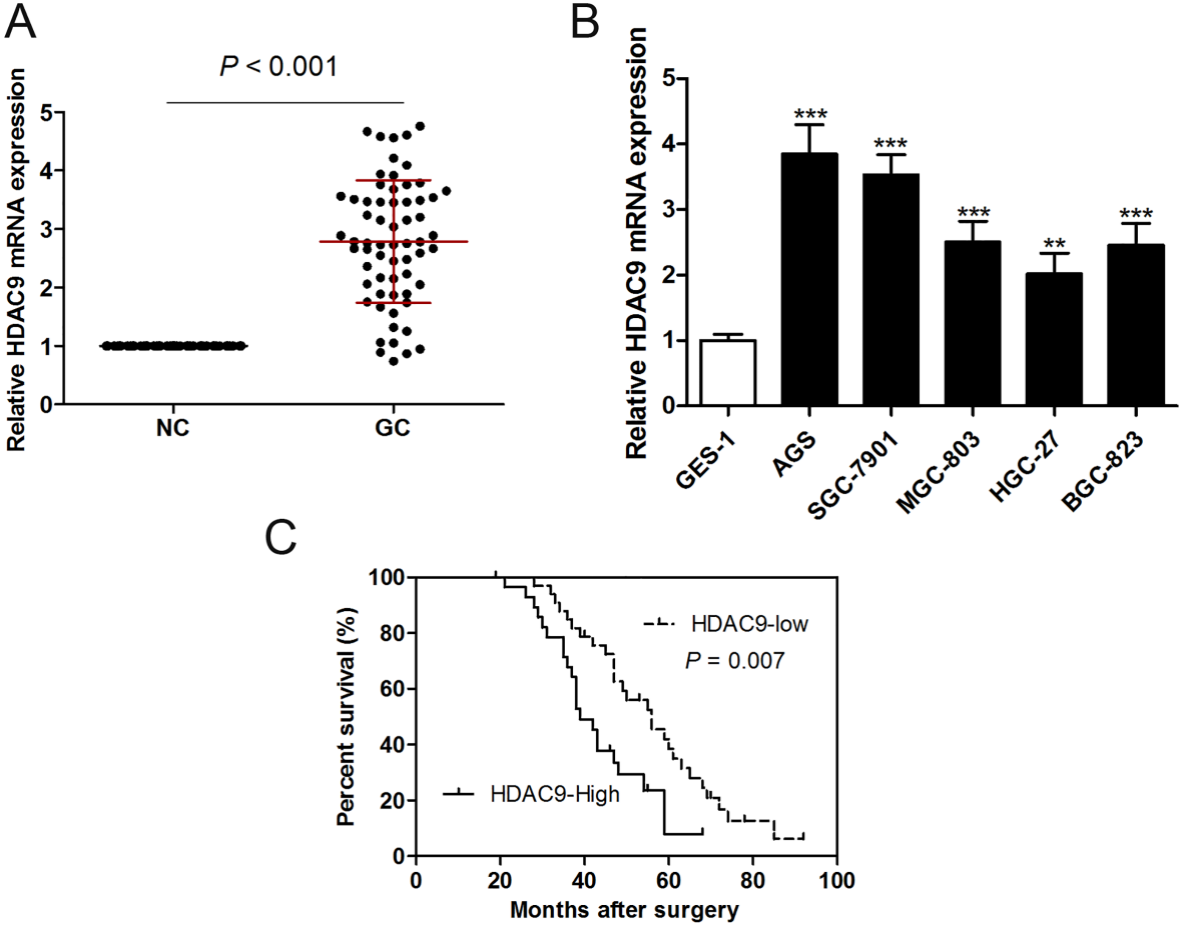
HDAC9 was up-regulated in gastric cancer (GC) tissues and cell lines and associated with poor prognosis. **A**, mRNA expression of HDAC9 in 63 pairs of GC tissues and corresponding non-tumorous tissues (NC). **B**, Human normal gastric epithelial cell line GES-1 and five GC cell lines AGS, SGC-7901, MGC-803, HGC-27, and BGC-823 measured by RT-qPCR. **C**, Kaplan-Meier analysis of the association between HDAC9 expression and overall survival in GC patients. Data are reported as means±SE. **P<0.01, ***P<0.001 compared with GES-1 group (ANOVA).

We first investigated the expression of HDAC9 in GC tissues, and the results demonstrated that HDAC9 mRNA expression was markedly increased in GC tissues compared with corresponding non-tumorous tissues (Figure 1A). We also found that HDAC9 mRNA expression was significantly higher in five GC cell lines than that of normal GES-1 cells (Figure 1B). Kaplan-Meier analysis revealed that high HDAC9 expression was associated with shorter overall survival prognosis (Figure 1C), poorer TNM stages, and lymph nodes metastasis (Table 1).

**Table 1.** Correlation between clinicopathological factors and HDAC9 expression levels in gastric cancer tissues.

Variable	Number of patients	HDAC9 (low)	HDAC9 (high)	P value
Gender				0.824
Male	39	20	19	
Female	24	13	11	
Age (years)				0.498
<60	35	17	18	
≥60	28	16	12	
Tumor size (cm)				0.593
<5	42	21	21	
≥5	21	12	9	
TNM stages				0.002
I-II	23	18	5	
III-IV	40	15	25	
Lymph nodes metastasis				<0.001
Negative	24	20	4	
Positive	39	13	26	

TNM: tumor, node, metastasis.

### HDAC9 knockout retarded cell proliferation and promoted apoptosis in GC cell lines

**Figure 2.**
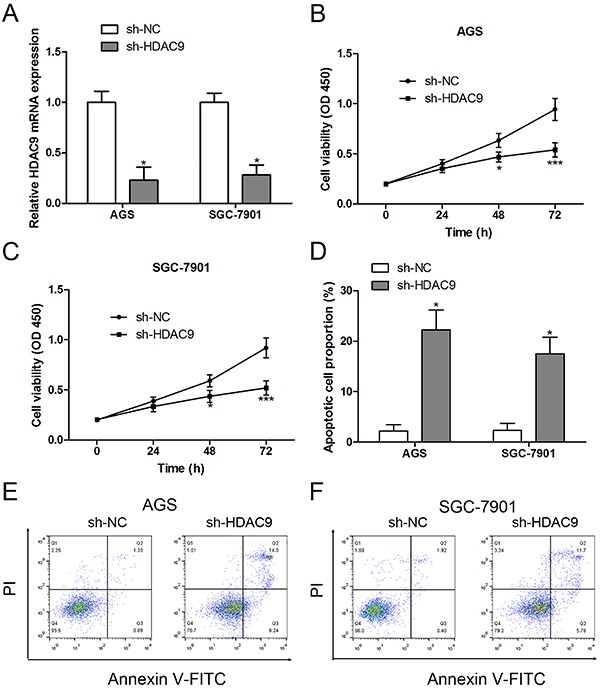
HDAC9 knockout retarded cell proliferation and promoted apoptosis in gastric cancer (GC) cell lines. **A**, After transfection with sh-NC (normal control) and sh-HDAC9, the mRNA expression of HDAC9 was measured by RT-qPCR. AGS (**B**) and SGC-7901 (**C**) cell viability were assessed by CCK-8 assay. Annexin V-FITC/PI double staining was performed to calculate cell apoptosis (**D**, **E**, and **F**). Data are reported as means±SE. *P<0.05, ***P<0.001 compared with control group; n=3 in each group (Student's *t*-test).

To investigate the pro-oncogenic role of HDAC9 in GC, we silenced HDAC9 expression by shRNA. After transfection with sh-HDAC9, the mRNA expression levels of HDAC9 were dramatically inhibited in AGS and SGC-7901 cells (Figure 2A). Further experiments revealed that HDAC9 knockout reduced the proliferation rate (Figure 2B and C) and caused an increase of apoptosis in AGS and SGC-7901 cells (Figure 2D, E, and F).

### HDAC9 was a direct target of miR-383-5p

**Figure 3.**
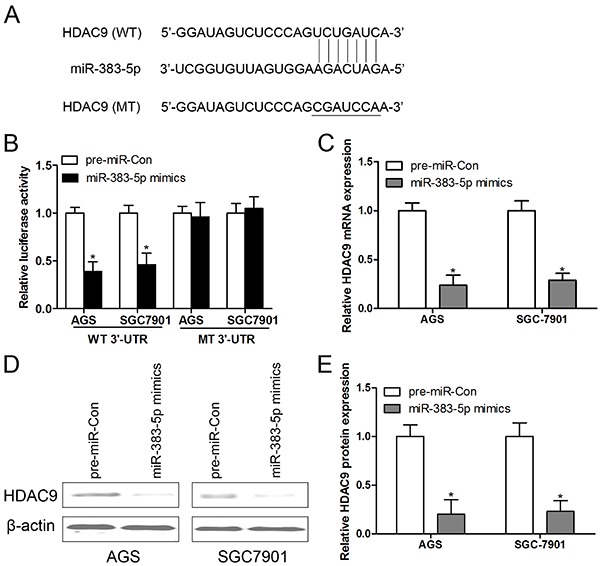
HDAC9 was a direct target of miR-383-5p. **A**, The conserved binding sites between miR-383-5p and HDAC9 were predicted by Targetscan. **B**, Relative luciferase activity of AGS and SGC-7901 cell co-transfection with miR-383-5p mimics and wild type (WT) 3′-UTR of HDAC9 or miR-383-5p mimics and mutant (MT) 3′-UTR of HDAC9. After transfection with miR-Con and miR-383-5p mimics, the mRNA (**C**) and protein (**D** and **E**) expression of HDAC9 were detected by RT-qPCR and western blotting, respectively. Data are reported as means±SE. *P<0.05 compared with control group; n=3 in each group (Student's *t*-test).

To further survey a possible mechanism to regulate HDAC9 expression, Targetscan (USA) was used to predict HDAC9-related miRNAs. We found that one conserved domain in the 3′-UTR of HDAC9 could bind with miR-383-5p, and the simulative construction drawing is shown in Figure 3A. To further explain the assumption, luciferase reporter assays were performed in AGS and SGC-7901 cells, and the results showed that miR-383-5p overexpression suppressed luciferase activities in HDAC9 WT 3′-UTR reporter constructs, whereas the effect was abolished when the mutation sequences were introduced into their binding sites (Figure 3B). Moreover, both the mRNA (Figure 3C) and protein (Figure 3D and E) levels of HDAC9 were reduced in AGS and SGC-7901 cells after transfecting with miR-383-5p mimics.

### miR-383-5p was down-regulated in GC tissues and cell lines, and was associated with poor prognosis

**Figure 4.**
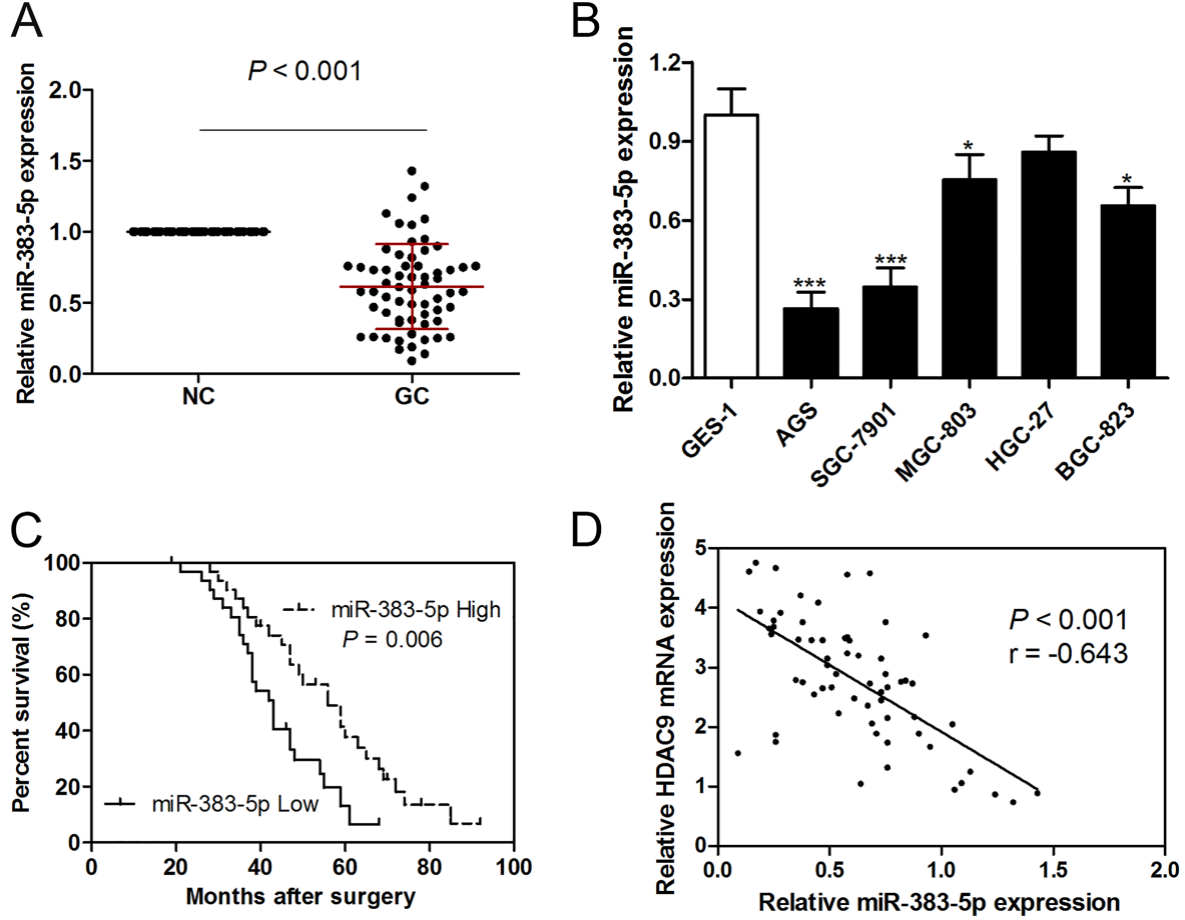
miR-383-5p was down-regulated in gastric cancer (GC) tissues and cell lines and associated with poor prognosis. **A**, Expression of miR-383-5p in 63 pairs of GC tissues and corresponding non-tumorous tissues (NC). **B**, Human normal gastric epithelial cell line GES-1 and five GC cell lines AGS, SGC-7901, MGC-803, HGC-27, and BGC-823 measured by RT-qPCR. **C**, Kaplan-Meier analysis of the association between miR-383-5p expression and overall survival prognosis in GC patients. **D**, Pearson's correlation analysis was used to evaluate the association between miR-383-5p and HDAC9 expression in 63 GC tissues. Data are reported as means±SE. *P<0.05, ***P<0.001 compared with GES-1 group (ANOVA).

The expression of miR-383-5p was investigated in 63 pairs of GC tissues and corresponding non-tumorous tissues. We observed that miR-383-5p expression was significantly decreased in GC tissues compared with corresponding non-tumorous tissues (Figure 4A). In addition, miR-383-5p expression was reduced in GC cell lines, except in HGC-27 cells (Figure 4B). Kaplan-Meier analysis revealed that low miR-383-5p expression was associated with shorter overall survival prognosis (Figure 4C), larger tumor size, poorer TNM stages, and lymph nodes metastasis (Table 2). Furthermore, Pearson's correlation analysis showed an inverse correlation between miR-383-5p and HDAC9 in 63 GC tissues (Figure 4D).

**Table 2.** Correlation between clinicopathological factors and miR-383-5p expression levels in gastric cancer tissues.

Variable	Number of patients	miR-383-5p (low)	miR-383-5p (high)	P value
Gender				0.256
Male	39	17	22	
Female	24	14	10	
Age (years)				0.535
<60	35	16	19	
≥60	28	15	13	
Tumor size (cm)				<0.001
<5	42	14	28	
≥5	21	17	4	
TNM stages				0.001
I-II	23	5	18	
III-IV	40	26	14	
Lymph nodes metastasis				0.048
Negative	24	8	16	
Positive	39	23	16	

TNM: tumor, node, metastasis.

### HDAC9 and miR-383-5p were independent prognostic factors of overall survival

The prognostic value of miR-383-5p and HDAC9 were evaluated using multivariate Cox proportional hazards regression analyses. Both miR-383-5p (HR=2.49, 95% CI=1.13−6.79, P=0.027) and HDAC9 (HR=2.82, 95% CI=1.26−7.53, P=0.019) were identified as independent prognostic factors for predicting overall survival in GC patients (Table 3).

**Table 3.** Univariate and multivariate regression analysis of gastric cancer patients for overall survival.

Variables	Univariate		Multivariate	
	HR (95% CI)	P value	HR (95% CI)	P value
Gender (female *vs* male)	0.89 (0.41–1.66)	0.681		
Age (≥60 *vs* <60)	0.95 (0.47–1.92)	0.797		
Tumor size (≥5 *vs* <5)	1.27 (0.54–3.01)	0.616		
TNM stages (III-IV *vs* I-II)	2.36 (1.33–4.57)	0.009	3.01 (1.43–6.01)	0.011
Lymph nodes metastasis (P *vs* N)	3.44 (1.59–6.93)	0.002	1.83 (0.77–3.91)	0.133
miR-383-5p (low *vs* high)	2.78 (1.39–5.71)	0.007	2.49 (1.13–6.79)	0.027
HDAC9 (high *vs* low)	3.15 (1.44–6.11)	0.004	2.82 (1.26–7.53)	0.019

TNM: tumor, node, metastasis; P: positive; N: negative; HR: hazard ratio.

### Overexpression of miR-383-5p inhibited cell proliferation and induced apoptosis in GC cell lines

**Figure 5.**
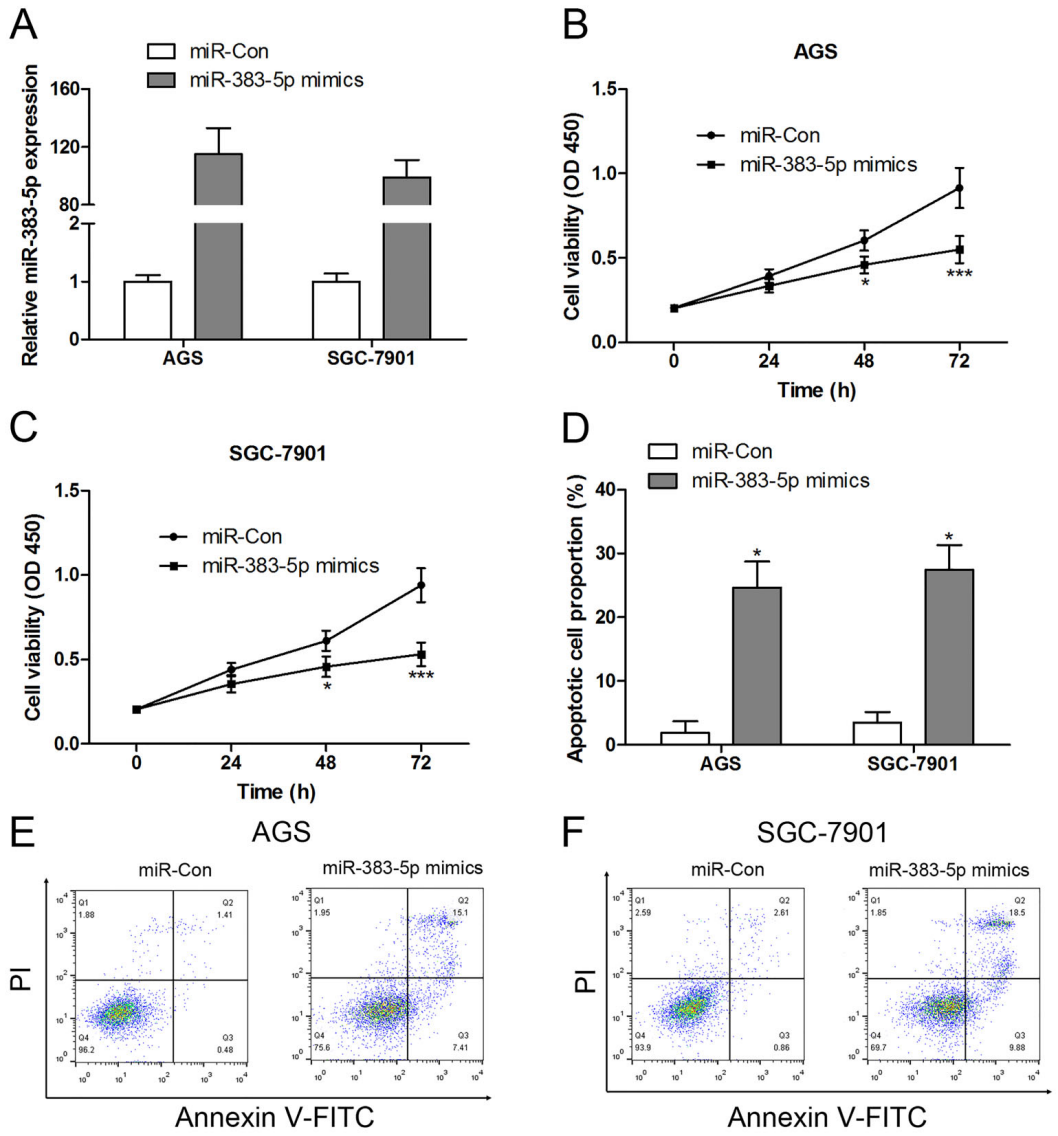
Overexpression of miR-383-5p inhibited cell proliferation and induced apoptosis in gastric cancer (GC) cell lines. **A**, After transfection with miR-Con or miR-383-5p mimics, the expression of miR-383-5p was measured by RT-qPCR. AGS (**B**) and SGC-7901 (**C**) cell viability was assessed by CCK-8 assay. Annexin V-FITC/PI double staining was performed to calculate cell apoptosis (**D**, **E**, and **F**). Data are reported as means±SE. *P<0.05, ***P<0.001 compared with control group; n=3 in each group (Student's *t*-test).

To elucidate the biological significance of miR-383-5p in gastric carcinogenesis, we first transfected with miR-383-5p mimics into AGS and SGC-7901 cells, and the expression of miR-383-5p was elevated in AGS and SGC-7901 cells (Figure 5A). Moreover, we observed that the overexpression of miR-383-5p inhibited cell proliferation (Figure 5B and C) and induced apoptosis (Figure 5D, E, and F) in AGS and SGC-7901 cells.

### Overexpression of miR-383-5p suppressed tumor growth of the GC cell xenograft in nude mice

**Figure 6.**
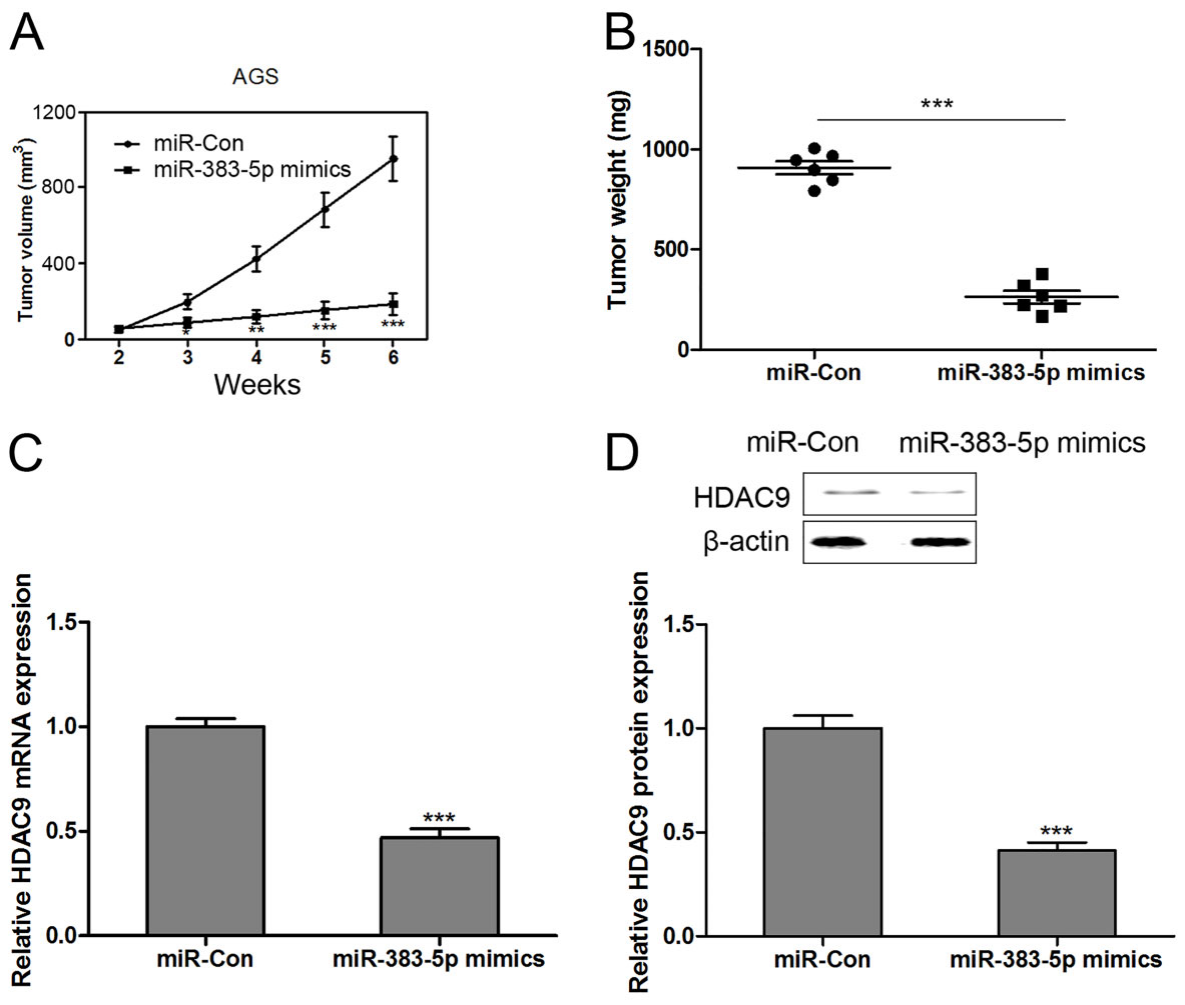
Overexpression of miR-383-5p suppressed the tumor growth of the gastric cancer (GC) cell xenograft in nude mice. AGS cells were transfected with miR-Con or miR-383-5p mimics, and then cells (1×10^7^ cells per 0.1 mL) were implanted subcutaneously into 4-week-old castrated male nude mice (n=6 in each group), and tumor volume and weight were evaluated at week 6 after cell implantation (**A** and **B**). The mRNA (**C**) and protein (**D**) expression of HDAC9 in solid tumor were detected by RT-qPCR and western blotting, respectively. Data are reported as means±SE. **P<0.01, ***P<0.001 compared with control group; n=6 in each group (Student's *t*-test).

To explore the role miR-383-5p in gastric carcinogenesis *in vivo*, the xenograft model of AGS cells in nude mice was carried out. AGS cells with stably expressed miR-383-5p mimics and blank control cells were injected subcutaneously into each flank of nude mice. We found that both tumor volume and weight were reduced by transfection with miR-383-5p mimics (Figure 6A and B). Compared with the blank control, miR-383-5p overexpression significantly suppressed the mRNA and protein levels of HDAC9 in the solid tumor from nude mice (Figure 6C and D).

## Discussion

In the present study, we found that HDAC9 was up-regulated in GC tissues and cell lines and closely related to poor prognosis and lymph nodes metastasis in GC patients. HDAC9 knockout led to inhibition of growth and an increase of apoptosis in AGS and SGC-7901 cells. More importantly, we demonstrated that miR-383-5p as a post-transcriptional regulator inhibited HDAC9 expression and was inversely correlated with HDAC9 expression in GC tissues. miR-383-5p had the opposite effects of HDAC9 on gastric carcinogenesis.

Recent studies have demonstrated both pro-oncogenic and tumor suppressive role for HDAC9 in different cancers (13,25–27). For example, HDAC9 expression levels are reduced in lung cancer cells, the restoration of its levels in lung cancer cells severely attenuates their growth activity *in vitro*, reflecting that HDAC9 may be a tumor suppressor and its downregulation promotes lung carcinogenesis (25). In contrast to that, HDAC9 is overexpressed in the tumor tissues from breast cancer (26), OSCC (13), and retinoblastoma (27), and inhibition of HDAC9 suppresses cancer cell proliferation, migration, and invasion (16,27,28). Moreover, some studies have noted that overexpression of HDAC9 is associated with poor prognosis and tumor progression of breast cancer and acute lymphoblastic leukemia (26,29). Our study revealed that HDAC9, as an oncogene, was up-regulated and associated with poor prognosis in patients with GC.

Numerous studies have suggested that miRNAs are important regulators in mediating oncogenic and tumor suppressive pathways and are estimated to target several hundred distinct genes in controlling various cancer processes (7 [Bibr B08] [Bibr B09],^13^ [Bibr B14],^19^ [Bibr B20],^21^). For example, miR-383-5p overexpression inhibits cell proliferation and enhances chemosensitivity in ovarian cancer cells by down-regulating tripartite motif containing 27 (30). miR-383-5p reverses hepatocellular carcinoma cell growth via targeting aldo-keto reductase family 1 member B10 (31). In our study, we showed that miR-383-5p functioned as a tumor suppressor in GC by inhibiting HDAC9 expression. Recently, Azarbarzin et al. confirmed the down-regulation of miR-383 in intestinal-type GC, and showed that miR-383 can serve as a diagnostic and prognostic biomarker in intestinal-type GC patients (23). Consistent with these findings, our results indicated that miR-383-5p was significantly decreased in GC tissues, and low miR-383-5p expression level was closely related with poorer prognosis in GC patients.

miR-383-5p targets to 235 transcripts with conserved sites, containing a total of 243 conserved sites and 83 poorly conserved sites (TargetScan 7.2 database; http:/www.targetscan.org), and is located on human chromosome 8p22, a region which has been found to be silenced in numerous cancer types, including ovarian cancer (30) and lung adenocarcinoma (32). Up-regulation of miR-383-5p suppresses proliferation and increases ovarian cancer cell apoptosis rate under treatment with paclitaxel (30). Moreover, overexpression of miR-383-5p in A549 and H1299 LAC cell lines inhibits cell proliferation by G1 cell cycle phase arrest and induction of apoptosis (32). Consistent with those findings, our experiments showed that over-expression of miR-383-5p inhibited cell proliferation and induced apoptosis in AGS and SGC-7901 cells *in vitro* and blocked solid tumor growth *in vivo.*

Previous studies have shown that several miRNAs target HDAC9 to exert their tumor repressive effect (12,13,33). For example, by reducing HDAC9 expression, miR-101-3p, miR-206, and miR-377 prevent the progression and development of retinoblastoma, breast cancer, and OSCC, respectively (12,13,33). In our study, we found that HDAC9 was a direct target of miR-383-5p, which was validated by bioinformatics tools and experimental validations. Over-expression of miR-383-5p decreased the expression levels of HDAC9 *in vivo* and *in vitro*. However, we deemed that the number of samples was a major limiting factor, which could lead to bias in our results. These findings would be more interesting if the prognostic value of miR-383-5p and HDAC9 were established in a larger sample size.

In conclusion, we showed for the first time that miR-383-5p was decreased in GC tissues and cell lines and served as a tumor suppressor by inhibiting HDAC9 expression to prevent gastric carcinogenesis. Our results suggested that miR-383-5p played an important role in gastric carcinogenesis, and it is one of the important mechanisms to regulate oncogenic HDAC9 in GC, which might be helpful in the development of novel therapeutic strategies for the treatment of GC.
